# Supermicrosurgical perforator-to-perforator flap reconstruction for lower limb defects: a single-center case series

**DOI:** 10.1080/23320885.2026.2740929

**Published:** 2026-10-19

**Authors:** Paneed F. Jalili, Joris E. Eeuwen, Laura Hilbig-Vlatten, Julius Asschenfeldt, Arriyan S. Dowlatshahi

**Affiliations:** a Division of Orthoplastic and Reconstructive Microsurgery, Beth Israel Deaconess Medical Center, Harvard Medical School, Boston, MA, USA; b Medical Faculty, University of Freiburg, Freiburg, Germany

**Keywords:** Supermicrosurgery, perforator-to-perforator anastomosis, lower extremity reconstruction, free flap, orthoplastic surgery

## Abstract

Lower limb soft tissue defects remain a major reconstructive challenge due to frequent exposure of critical structures and compromised vascularity. In complex cases where local tissue is insufficient, free tissue transfer is required. Advances in supermicrosurgery have enabled perforator-to-perforator (P-to-P) anastomoses allowing reliable perfusion while preserving major axial vessels and minimizing donor-site morbidity. A retrospective review was conducted of five patients who underwent P-to-P free flap reconstruction for lower limb defects between January 2018 and January 2023. Preoperative CT angiography and intraoperative duplex ultrasound were used for perforator mapping. Two anterolateral thigh, one medial sural artery perforator, and two gracilis muscle flaps were utilized. Anastomoses were hand-sewn using 10-0 to 12-0 nylon under high-magnification microscopy. Flap perfusion was monitored with Vioptix Tissue Oximeter. The mean age was 60.8 years and defect sizes ranged from 5 × 4 cm to 20 × 8 cm. All flaps survived without partial or total loss. One patient developed minor wound dehiscence that healed without intervention. Reconstruction was successful across varied etiologies including infection, chronic wounds, and postoperative defects, with low morbidity and no re-explorations. P-to-P supermicrosurgical reconstruction offers a reliable, vessel-preserving approach for complex lower limb defects. This technique maintains major vascular axes, minimizes donor-site morbidity, and achieves outcomes comparable to conventional microsurgical methods across diverse etiologies such as infection, chronic wounds, and postoperative defects. In our series, flap survival was universal with no re-explorations and minimal morbidity. Wider clinical adoption and continued experience may further define its role in contemporary lower extremity reconstruction

## Introduction

Lower limb soft tissue defects pose a significant reconstructive challenge due to their complex anatomy, high functional demands, and frequent exposure of critical structures such as bone, tendons, or hardware. These defects commonly result from trauma, infection, chronic non-healing wounds, degenerative conditions, or oncologic resection [1]. When local tissue is insufficient for durable coverage, free tissue transfer becomes essential to achieve wound closure and preserve limb function [2].

Supermicrosurgery, defined as the anastomosis of vessels measuring 0.3 to 0.8 mm in diameter, has expanded the reconstructive armamentarium by enabling precise dissection and anastomosis of very small-caliber perforators [3]. This advancement allows for tissue transfer that minimizes donor-site morbidity and preserves major vascular axes, offering a vessel-sparing solution particularly suited to the lower extremity.

Patients requiring lower limb reconstruction often present with comorbidities that impair peripheral circulation, such as diabetes mellitus and peripheral vascular disease. Vascular compromise secondary to trauma or prior amputation further complicates reconstruction, as damaged recipient vessels heighten the risk of flap failure [4]. A recent systematic review and meta-analysis on supermicrosurgical lower limb reconstruction reported a high prevalence of comorbidities within this patient population, with 43.0% having diabetes mellitus, 14.5% peripheral vascular disease, and 12.0% hypertension, underscoring the clinical complexity and reconstructive challenges inherent to these cases [4].

In response to these challenges, reconstructive strategies have evolved from conventional free flap techniques toward refined, perforator-based transfers. Advances in high-resolution microscopes, specialized instruments, and ultra-fine sutures have facilitated the development of perforator-to-perforator (P-to-P) flaps, a supermicrosurgical technique that enables anastomosis between small perforating vessels^[^^5^^]^. This approach preserves major axial vessels, reduces donor and recipient site morbidity, and lowers the risk of major vessel injury [6,7].

In this case series, we present our institutional experience with P-to-P flap reconstruction for lower limb defects. We aim to describe the surgical technique, patient selection, and postoperative outcomes, and to clarify the role and advantages of supermicrosurgical reconstruction in complex lower extremity reconstruction.

## Patients and methods

From January 2018 to January 2023, a total of five patients underwent P-to-P reconstruction for lower limb defects of varying etiologies. A variety of perforator flaps were used, all utilizing a supermicrosurgical end-to-end anastomosis technique. P-to-P anastomosis was selected in these cases because it allows for more limited dissection of both donor and recipient vessels, facilitating a more expedient and less invasive reconstruction. Additionally, in patients with significant peripheral arterial disease and calcified major vessels, distal perforators are often relatively spared from atherosclerotic involvement, making them more suitable recipient targets.

All patients received CT angiograms preoperatively to identify vessel targets. Preoperatively, duplex ultrasound was used to pinpoint the location of the recipient perforators, anticipating the anastomosis location.

The study was approved by the Institutional Review Board (approval no. 2023P000743) and conducted in accordance with the Declaration of Helsinki. Written informed consent to publish clinical details and images was obtained from all subjects. 

### Surgical technique

The patient is placed in supine positioning on the operating room table with the relevant limb outstretched, prepped circumferentially with Betadine, and draped in standard fashion. The defect is debrided in an excisional fashion down to healthy bleeding tissue, and the total dimensions of the defect are measured. The recipient vessels are prepared by exsanguinating the extremity and elevating a tourniquet. The appropriate vessel(s) are carefully identified, dissected, mobilized, and a microclamp is placed in anticipation of anastomosis. Upon release of the tourniquet, the selected perforator is confirmed as pulsatile and of adequate caliber.

Attention is then turned to the donor site, where a template of the wound was transferred, and appropriate perforators are identified. The required length of the pedicle is predicted. The flap is incised and islandized.

The flap is then detached, flushed with a heparinized saline solution, and brought to the recipient site. A hand-sewn P-to-P anastomosis is performed for both arterial and venous connections. We used 11-0 and 12-0 microsuture with 50- to 75-μm needles and an operating microscope. Appropriate inflow and outflow were confirmed after reperfusion of the flap, using clinical criteria (bleeding from dermal edge or muscle), vein and artery stripping, Doppler, and ViOptix Tissue Oximetry (Vioptix Inc., Fremont, CA, USA), a near-infrared spectroscopy device that continuously measures flap oxygen saturation to assess perfusion in real time for skin flaps.

## Results

Five patients were included in this series, four were male and one was female, with a mean age of 60.8 years (standard deviation: 17.8 years). The mean BMI was 26.3 kg/m^2^ (standard deviation: 3.1 kg/m^2^) (Table 1). Defect locations included the left foot, right distal tibia, right heel, left ankle, and left distal tibia. Defect dimensions ranged from 5 × 4 cm to 20 × 8 cm. Flap types used for reconstruction were the anterolateral thigh (ALT) flap in two cases, medial sural artery perforator (MSAP) flap in one case, and gracilis muscle flap in two cases. All reconstructions involved end-to-end microvascular anastomosis using 10-0 or 11-0 nylon sutures (Table 2 and 3).

**Table 1.** Patient demographics.

**Age**	60.8 years ± 17.8 years
**BMI**	26.3 kg/m² ± 3.1 kg/m²
**Sex**	
Male	4
Female	1
**Race/Ethnicity**	
White	3
Black	1
Hispanic	1

**Table 2.** Defect and reconstruction characteristics.

Case	Defect location	Defect dimensions (cm)	Flap used	Recipient perforator	Suture size
**1**	Left foot	15 × 7.5	ALT (L)	Perforating branch of dorsalis pedis artery	9–0 to 11–0
**2**	Right distal tibia	20 × 8	ALT (L)	Soleus perforators from posterior tibial system	10–0
**3**	Right heel	9 × 4	MSAP (R)	Posterior tibial artery perforator	9–0 to 10–0
**4**	Left ankle	5 × 5	Gracilis (R)	Lateral tarsal artery	9–0 to 10–0
**5**	Left distal tibia	10 × 6	Gracilis (R)	Posterior tibial artery perforator	10–0

**Table 3.** Anastomotic characteristics and operative details.

Case	Operative time	Documented arterial diameter	Size mismatch	Technical adjustments
**1**	7 h 08 min	Not documented	Venous mismatch	Venous tapering
**2**	11 h 31 min	Approx. 0.5–1.2 mm after revision	Initial 2:1 arterial mismatch	Revision to dual P-to-P arterial anastomoses
**3**	5 h 50 min	Approx. 0.7 mm recipient perforator	Venous mismatch	Venous tapering and additional venous anastomosis
**4**	4 h 38 min	Approx. 1.0 mm recipient artery	Flap vein approx. 3 times recipient vein	Hand-sewn venous tapering
**5**	3 h 56 min	Approx. 1.0–1.2 mm	Minimal arterial mismatch	1.5 mm venous coupler

### Post-operative management

ViOptix tissue oximeter was used for flap monitoring postoperatively until postoperative day 5, when the patient is discharged. Trends of ViOptix were utilized, noting decreases of greater than 20 percentage points from baseline, which would prompt return to the operating room for exploration.

### Complication and outcomes

In one patient, distal wound dehiscence was reported 9 days postoperatively, which did not require additional treatment and healed without intervention. No revision surgeries were required following reconstruction, and no partial or total flap losses occurred. Median follow-up duration was 14.3 months (range, 9.1–39.7 months). At most recent follow-up, no additional short- or long-term complications were identified.

### Case demonstrations

Case 1: *Left ALT flap with P-to-P anastomosis to left foot, superficial peroneal-lateral femoral nerve transfer, and dual lymphatic venous anastomosis.*

A 54-year-old male with non-insulin-dependent diabetes mellitus (A1C 7.9%) previously underwent excision of an unidentified soft-tissue mass on the left lateral ankle, resulting in a 15 × 5 cm wound. After thorough debridement, the final defect measured 15 × 7.5 cm. During flap elevation, multiple side branches were identified along the intramuscular course of the perforator. This complex anatomy guided the decision to employ a P-to-P flap to preserve perfusion to the vastus lateralis. A meticulous hand-sewn end-to-end anastomosis was performed between a perforator of the lateral femoral circumflex artery and a perforating branch of the dorsalis pedis artery using 11-0 nylon.

The superficial peroneal nerve demonstrated a neuroma at the defect site. Neurolysis of the lateral femoral cutaneous nerve was performed, and the resected neuroma was reconstructed with an end-to-end neurorrhaphy using 8-0 nylon, reinforced with a nerve conduit and fibrin sealant (Tisseel, Baxter, Deerfield, IL). Two lymphaticovenous anastomoses were subsequently created using 10-0 nylon to address associated lymphatic compromise (Figure 1).

**Figure 1.**
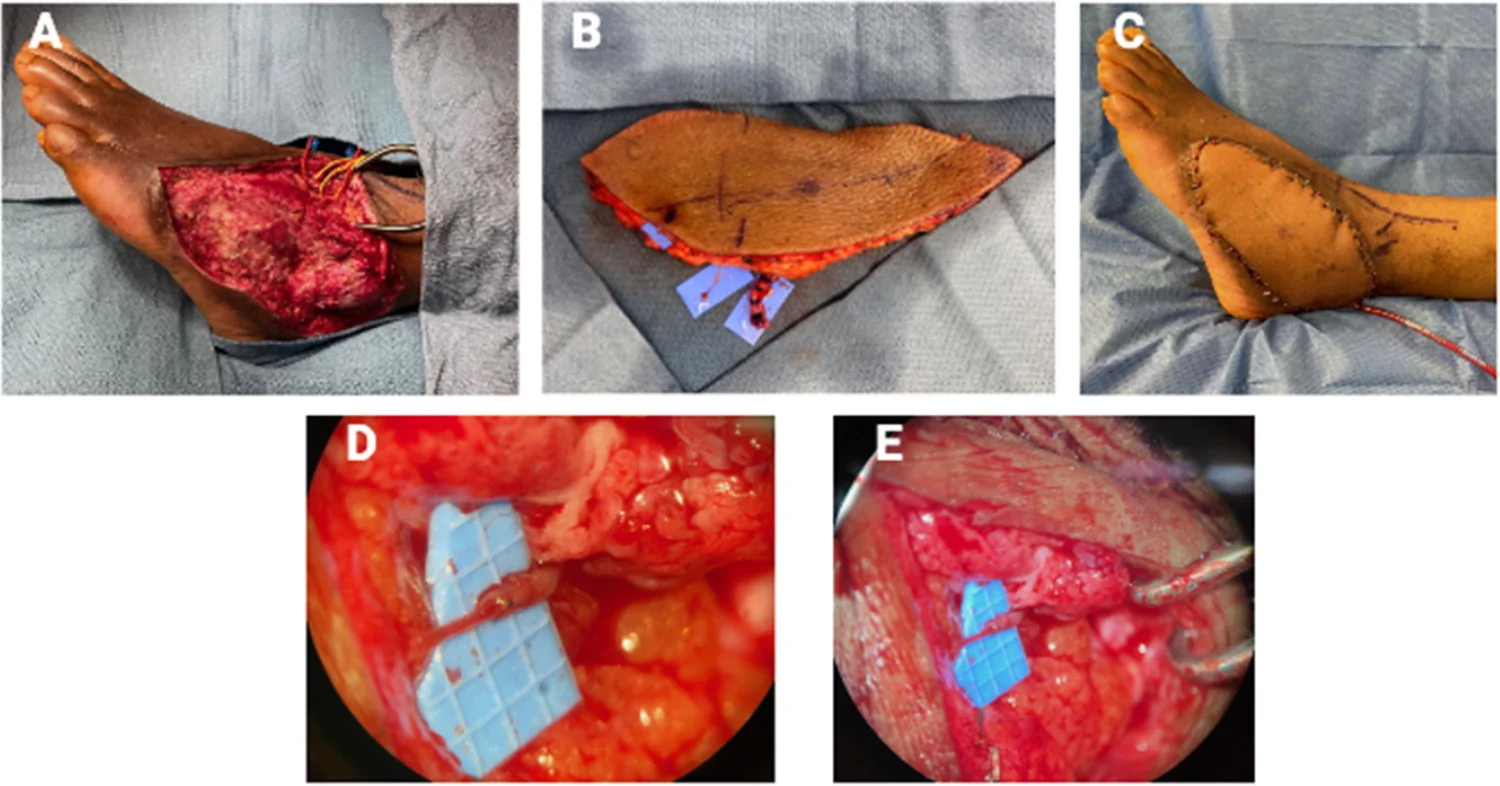
(A) Recipient site, (B) Harvested ALT flap, (C) Postoperative view demonstrating inset and flap viability. (D, E) Microsurgical anastomosis.

Case 2: *Left ALT with P-to-P anastomosis to right distal tibia, and saphenous-lateral femoral cutaneous nerve transfer via vein graft*

A 53-year-old female with a history of open right distal tibia and fibula fracture had undergone multiple operations for tibial nonunion. Following a recent open reduction and internal fixation (ORIF) with a calcaneal bone graft, she developed wound dehiscence over the distal tibia. After debridement, the defect measured 20 × 8 cm.

During flap elevation, atherosclerotic plaque was noted along the descending branch of the lateral circumflex femoral artery, which was of larger caliber than the recipient vessel. Despite the vessel’s friable quality, an initial end-to-end anastomosis between the lateral circumflex femoral artery and a perforating branch of the posterior tibial artery was attempted but failed after reperfusion. Consequently, two P-to-P anastomoses were performed, connecting paired perforators of the lateral circumflex femoral and posterior tibial arteries using 10-0 nylon under supermicrosurgical technique.

For nerve reconstruction, the proximal stump of the saphenous nerve was coapted to the flap’s lateral femoral cutaneous nerve using a venous conduit fashioned from an autologous saphenous vein graft (Figure 2).

**Figure 2.**
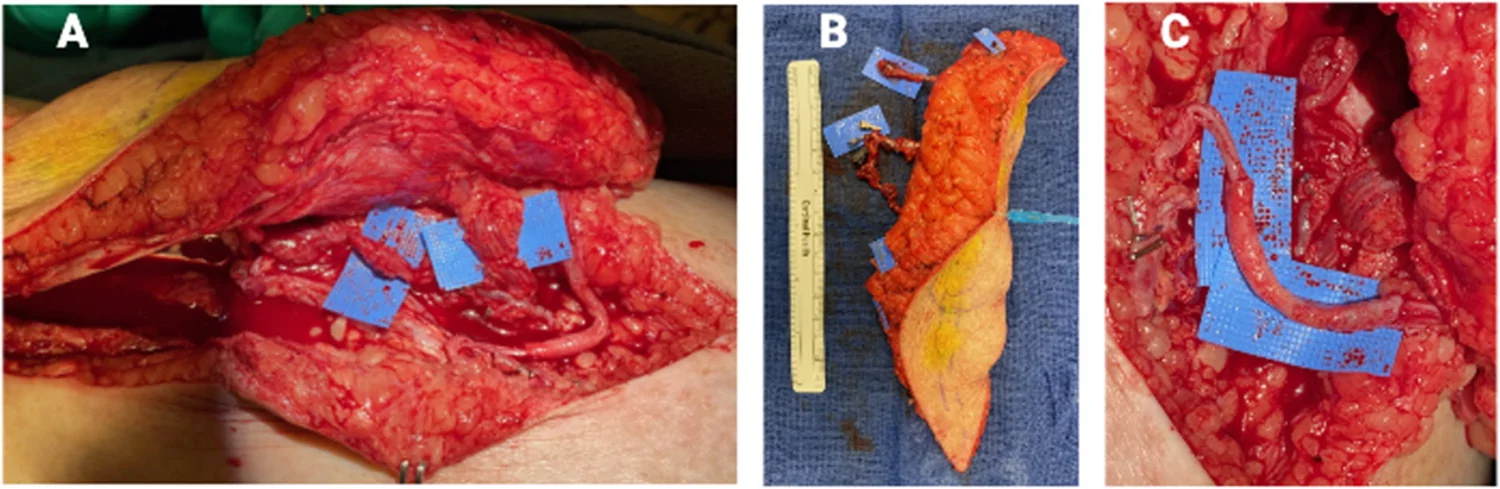
(A) Donor site with perforators, (B) Harvest ALT flap, and (C) Microsurgical anastomosis.

Case 3: *Right MSAP with P-to-P anastomosis to right heel*

A 73-year-old male who had undergone right Achilles tendon repair two months earlier presented with a persistent non-healing wound over the posterior ankle with exposed Achilles tendon. After surgical debridement, the defect measured 9 × 4 cm.

A supermicrosurgical end-to-end anastomosis was performed between the MSAP and a perforating branch of the posterior tibial artery to minimize dissection at both the donor and recipient sites, using 10-0 nylon. The larger of the two venous pedicles was anastomosed end-to-end with a corresponding posterior tibial vein using the same suture caliber (Figure 3).

**Figure 3.**
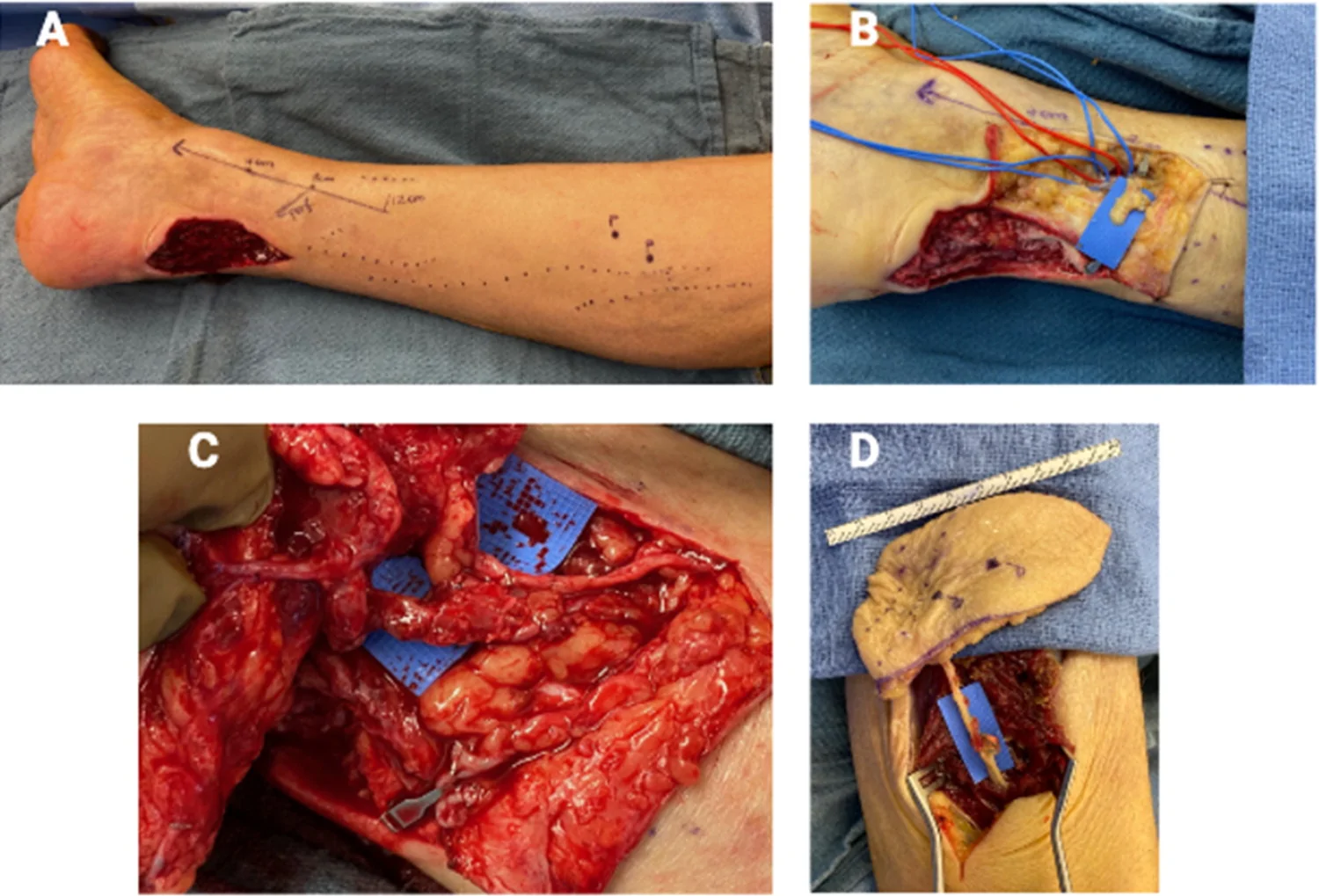
(A) Defect and preoperative markings, (B) Recipient site peroneal perforator, (C) Donor site, and (D) Elevated MSAP flap.

Case 4: *Right gracilis muscle flap with P-to-P anastomosis to left ankle, and TMR of the superficial peroneal- to gracilis motor nerve.*

A 37-year-old otherwise healthy male sustained bilateral ankle fractures following a fall from height. After open reduction and internal fixation (ORIF) of both ankles, he developed hardware infections. The right ankle was previously reconstructed with an ALT flap. Given the patient’s hypercoagulable state and the increased vascular resistance noted during the prior reconstruction, a muscle flap was selected for its lower resistance characteristics.

To preserve primary vascular routes and mitigate the risk of vasospasm, a P-to-P anastomosis was performed between a gracilis muscle perforator and a perforating branch of the lateral tarsal artery using 10-0 nylon. Two venous anastomoses were created, one with the dorsalis pedis vein and another with a superficial vein, using 9-0 and 10-0 nylon, respectively. The superficial peroneal nerve was found to have a neuroma, which was excised, followed by end-to-end coaptation of the nerve to the gracilis motor nerve using 8-0 nylon, achieving targeted muscle reinnervation (TMR) (Figure 4).

**Figure 4.**
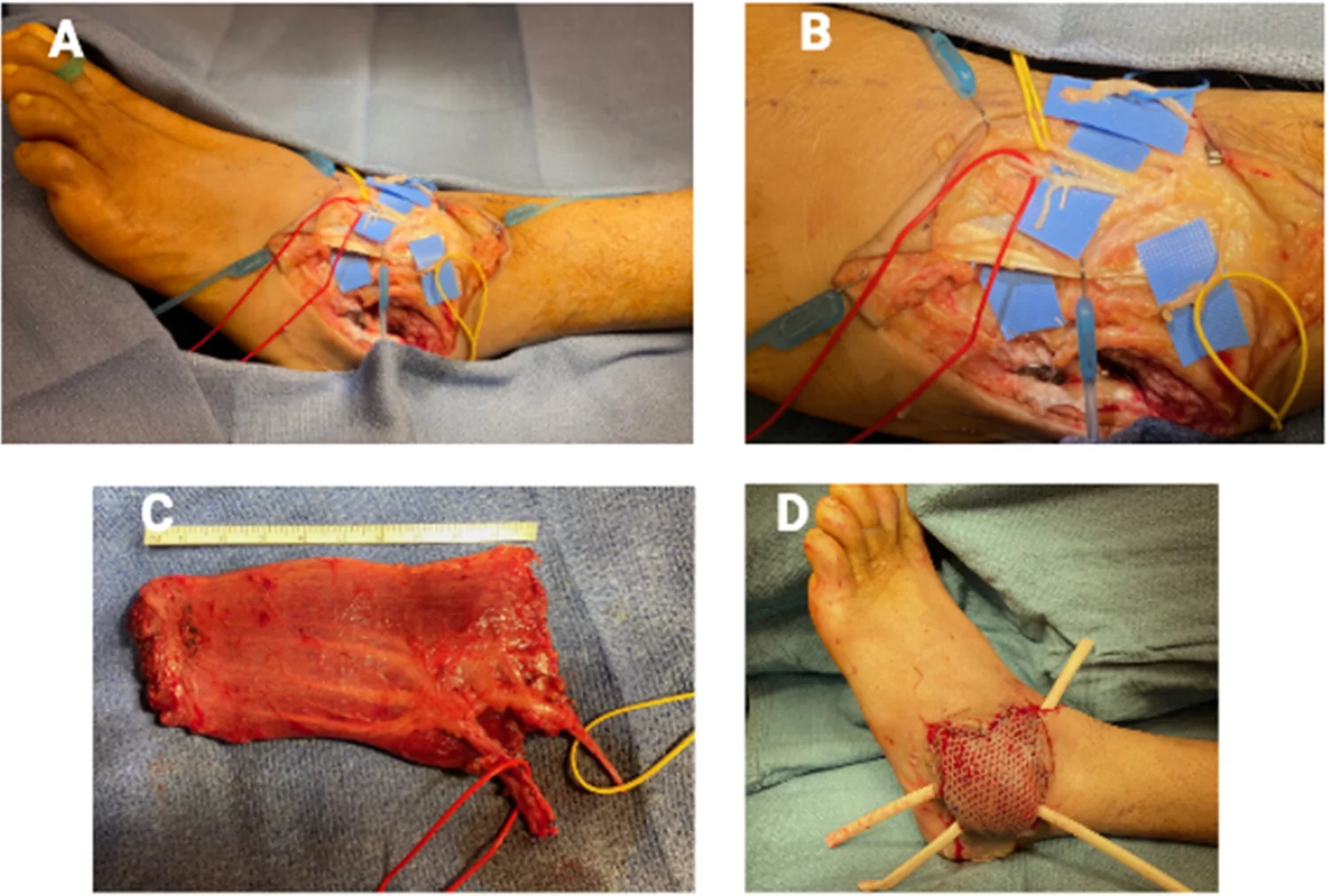
(A, B) Recipient site with isolated neurovasculature, (C) Harvested gracilis muscle flap, and (D) Postoperative view of flap inset and viability.

Case 5: *Right gracilis muscle flap with P-to-P anastomosis to left distal tibia*

An 83-year-old male with a history of atrial fibrillation, peripheral vascular disease, and a prior tibial fracture treated with an intramedullary nail developed a non-healing wound over the left shin following screw removal and irrigation and debridement (I&D). Initial debridement was performed, followed by application of negative pressure wound therapy.

Given the patient’s extensive peripheral vascular disease, a P-to-P approach was selected to avoid the risks associated with conventional anastomosis to major recipient vessels. This strategy was further supported by the patient’s diffuse atherosclerotic burden, rendering the case more technically challenging than a standard free tissue transfer.

After the second debridement, the defect measured 10 × 6 cm. An end-to-end anastomosis was performed between a gracilis muscle perforator and a perforating branch of the posterior tibial artery, approximately 15 cm proximal to the medial malleolus, using 10-0 nylon. Venous outflow was established with a 1.5 mm coupler (Figure 5).

**Figure 5.**
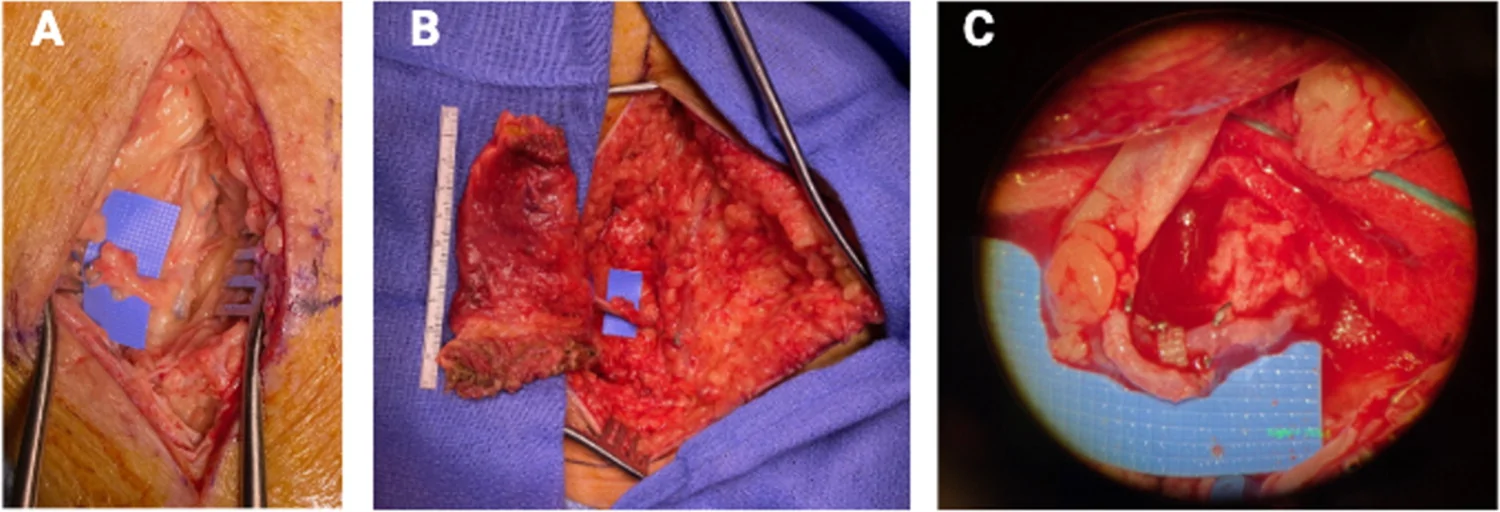
(A) Recipient site vessel, (B), Donor site gracilis flap harvest, and (C) Microsurgical anastomosis.

## Discussion

Lower extremity reconstruction remains a technically demanding domain within microsurgery, particularly in patients with compromised vascularity, prior reconstructive procedures, or limited donor options. The evolution toward less invasive, vessel-preserving strategies has culminated in the adoption of the P-to-P concept, which leverages the use of small-caliber, healthy distal vessels within the zone of injury. This approach minimizes donor-site morbidity and preserves major axial vessels, aligning with the broader trend toward supermicrosurgical techniques.

Recent institutional and multicenter experiences have demonstrated that P-to-P anastomosis provides reliable coverage for complex lower limb defects, with flap survival and complication rates comparable to those achieved with conventional free flap techniques. In a large cohort, Power et al. reported no significant difference in total or partial flap failure rates between perforator and major artery recipients, with the added benefit of shorter operative times when using perforator recipients [8]. Systematic review and meta-analysis further support the safety and efficacy of supermicrosurgical P-to-P reconstruction, with overall flap complication rates in line with traditional microsurgical approaches [4]. These findings are echoed in single-center series, which highlight the versatility and low morbidity of perforator-based flaps in a range of clinical scenarios, including trauma, tumor, and chronic wounds [9–11].

The P-to-P technique is particularly advantageous in high-risk populations, such as those with diabetes mellitus, peripheral vascular disease, or previous vascular interventions, where major vessels may be unsuitable for anastomosis [12,13]. However, the technical demands of supermicrosurgery necessitate a steep learning curve and careful patient selection to optimize outcomes [13].

The limitations of this study include its small sample size and retrospective design, which limit generalizability.

## Conclusion

P-to-P supermicrosurgical reconstruction represents a reliable and vessel-preserving option for complex lower extremity defects. In our series, this approach enabled successful limb salvage with 100% flap survival, no re-explorations, and minimal morbidity, even in patients with significant vascular comorbidities. By avoiding manipulation of major axial vessels and limiting dissection within scarred or diseased vascular territories, P-to-P anastomosis expands reconstructive options in high-risk patients. Although technically demanding, this technique offers a valuable addition to the modern orthoplastic armamentarium and warrants continued application and study in larger cohorts.

## Data Availability

The data supporting the findings of this study are available from the corresponding author upon reasonable request. Data are not publicly available due to patient privacy considerations.
